# BraX-Ray: An X-Ray of the Brazilian Computer Science Graduate Programs

**DOI:** 10.1371/journal.pone.0094541

**Published:** 2014-04-11

**Authors:** Luciano A. Digiampietri, Jesús P. Mena-Chalco, Pedro O. S. Vaz de Melo, Ana P. R. Malheiro, Dânia N. O. Meira, Laryssa F. Franco, Leonardo B. Oliveira

**Affiliations:** 1 School of Arts, Sciences and Humanities, University of São Paulo, São Paulo, SP, Brazil; 2 Center for Mathematics, Computation and Cognition, Federal University of ABC, Santo André, SP Brazil; 3 Computer Science Department, Federal University of Minas Gerais, Belo Horizonte, MG, Brazil; 4 Institute of Computing, University of Campinas, Campinas, SP, Brazil; 5 Institute of Computing, Fluminense Federal University, Rio de Janeiro, RJ, Brazil; 6 Faculty of Electrical Engineering and Computing, University of Campinas, Campinas, SP, Brazil; University of Cape Town, South Africa

## Abstract

Research productivity assessment is increasingly relevant for allocation of research funds. On one hand, this assessment is challenging because it involves both qualitative and quantitative analysis of several characteristics, most of them subjective in nature. On the other hand, current tools and academic social networks make bibliometric data web-available to everyone for free. Those tools, especially when combined with other data, are able to create a rich environment from which information on research productivity can be extracted. In this context, our work aims at characterizing the Brazilian Computer Science graduate programs and the relationship among themselves. We (i) present views of the programs from different perspectives, (ii) rank the programs according to each perspective and a combination of them, (iii) show correlation between assessment metrics, (iv) discuss how programs relate to another, and (v) infer aspects that boost programs' research productivity. The results indicate that programs with a higher insertion in the coauthorship network topology also possess a higher research productivity between 2004 and 2009.

## Introduction

Productivity assessments of research groups are increasingly relevant. There are limited funds to foment research activities and a strong competitiveness to obtain part of it. An accurate research productivity assessment would make possible to allocate funds meritocratically. The problem is that research productivity assessment is indeed a daunting task. This is because it involves both qualitative and quantitative analysis of several characteristics, most of them subjective in nature. Besides, there is no consensus on the metrics to be used in the analysis and then, depending on the chosen ones, the assessment may produce quite different results.

What sharply distinguishes Brazil from other countries is its natural and cultural diversity [1]. This diversity can also be found in science: there are a number of high standard graduate programs with unique characteristics spread throughout the territory. This is particularly true in the field of Computer Science (CS) since there are graduate programs of excellence in all of the five regions of the country. On one hand, these peculiarities makes hardly possible to characterize and assess programs. On the other hand, Brazil is one of few nations to have information on virtually all its publications openly available in the *World Wide Web* combined in a single web-based system: The Lattes Platform.

Therefore, by getting together Lattes, other web-based tools (e.g. Microsoft Academic Search and Google Scholar), and information accessible online about journal impact factors (e.g. Thompson's Journal Citation Reports and Scimago Journal Rank impact factors), it is possible to create a rich environment of raw data from which information can be extracted to characterize graduate programs as well as the relationship among themselves.

Although there is no consensual and precise measure for the complete analysis of graduate programs, some metrics are commonly used [2], namely: (i) programs' goals; (ii) faculty members; (iii) students; (iv) intellectual production; and (v) social insertion. These metrics are present in the graduate programs evaluation reports performed by the Brazilian Coordination for the Improvement of Higher Education Personnel (CAPES) once every three years, when CAPES assigns a weight (numerical value between 3 and 7) to each of them. According to CAPES, a program which weight is 7 excels in their respective fields worldwide. (CAPES is a public agency within the Brazilian Ministry of Higher Education).

Table 1 presents the Brazilian CS graduate programs whose CAPES weight is either 6 or 7.

**Table 1:** Top Brazilian CS Graduate Programs according to CAPES.

Prog. #	University	Institution/Department
1	PUC-RIO	Department of Informatics
2	UFMG	Computer Science Department
3	UFRJ	ALC Inst. and G. Sch. of Res. and Eng.
4	UFPE	Center of Informatics
5	UFRGS	Institute of Informatics
6	UNICAMP	Institute of Computing
7	USP/SC	Institute of Math. Science and Comp.

### Contribution

Our work aims at characterizing the Brazilian CS graduate programs and the relationship among themselves. To be precise, we (i) present views of the programs from different perspectives, (ii) rank the programs according to both each perspective and to a combination of them, (iii) show the correlation between assessment metrics, (iv) discuss how programs relate to another, and (v) infer aspects that boost programs' research productivity. To do that, mainly two characteristics are explored: (i) the intellectual productivity in terms of bibliographic production and (ii) the relationships among programs in terms of academic social networks. Quantitative indices (e.g. citation count as well as h- and g-index) that reflect the quality of intellectual output were associated with those two characteristics. Besides, all information used in the characterization is publicly available in the web.

The relevance of our work rests on the benefits of exploring different metrics and relationships to characterize the research productivity of programs. To our knowledge, ours is the first work to look at the Brazilian graduate programs from so many perspectives derived from either bibliographic productions or academic social networks.

## Materials and Methods

**Figure 1:**
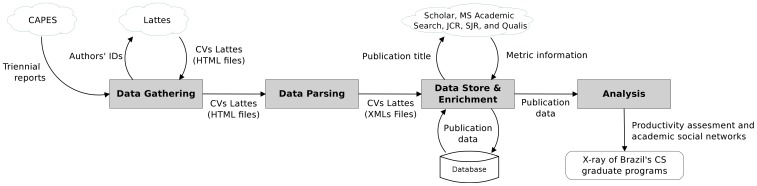
Schematic data flow diagram of the proposed method. Web-available data sources are represented by clouds. Processes are represented by blocks in gray color. Each arrow represents the information flow between processes/data sources.

In this work, we have assessed all the 37 existing CS academic graduate programs in Brazil presented in both 2004–2006 and 2007–2009 triennia. (Note we have not considered professional graduate programs in the study.) The evaluation has been carried out based on bibliographic production of professors of the programs during the period in question. Those professors have been identified via CAPES' reports and the list has been manually validated through the use of the Brazilian National Form of Graduate Programs (www.capes.gov.br/avaliacao/documentos-de-area-/3270). Both the reports about the Brazilian graduate programs and the curricula of the professors are openly-available in the Web as HTML files. For each professor, we have acquired her/his Lattes curriculum and extracted her/his full bibliographic productions published in academic journals and conferences. Based on this information, we have generated the academic statistics. The dataset contained 732 professors, 17,976 publications (13,926 conference papers and 4,050 journal papers), 7,583 co-authorship relationship pairs among professors, and 1,428 co-authorship relationship pairs among graduate programs. Figure 1 illustrates a schematic data flow diagram considered in our work. In what follows, we detail this method, describe tools employed, and discuss statistics used.

### Data Gathering

Lattes is a web-based curriculum system that embraces the curricula (i.e., research productivity) of the major professionals and researchers working in Brazil. Lattes curricula have been designed to show individual public information for every research registered on the system. In this context, performing a summarization and evaluation of bibliographic production for a group of registered researchers requires a systematic effort.

In this work, we have used the bibliographic production registered on Lattes as our data source. To gather this information, we first obtained programs' professor names from two CAPES's triennial reports (2004–2006 and 2007–2009). These reports, also called in Portuguese *Cadernos de Indicadores*, and other CAPE's documents are available in the web.

With this process, 732 professors were identified and associated with 37 CS academic graduate programs. These 37 programs are the ones presented in both triennia.

For each professor, we have gotten her/his corresponding Lattes ID (each ID is composed of 16 digits), which in turn make us able to build the URL to access her/his curriculum online. Thus, a curriculum in HTML format was obtained for each professor identified in the above process. This job has been carried out in a semi-automatic fashion through the Lattes searching tool (qualis.capes.gov.br/webqualis).

### Data Parsing

The HTML file from each curriculum is processed in two stages, namely (i) pre-processing and (ii) data extraction. The former removes the end-of-line characters, special characters and multiple blank spaces. This pre-processing is required to make easier the identification of patterns – which is performed in the next step. The latter is responsible for extracting relevant information from each curriculum.

To perform this extraction, a set of regular expressions (*regex*) is executed. Initially, the expressions are used to extract general and personal information, e.g., researcher's name and gender. Later on, a regex is used to break the curriculum into its main sections – e.g., *Academic Degrees*; *Research Areas of Interest*; and *Bibliographic Production*.

Subsequently, for each section, we have employed tailor-made regex to extract information precisely, e.g., in the *Bibliographic Production Section*, there are regular expressions to identify each sort of production, such as journal or conference papers. All items from each section is identified and organized according to their fields; for instance, journal paper description comprises the following fields: authors, title, publication year, page numbers as well as journal name, number, and volume. All information considered relevant is stored in an XML file, one from each curriculum. These files have then been used to populate a local relational database.

### Data Storage & Enrichment

We have set up a relational database and automatically populated it with data from the XML files computed in the above process. Like any other source of data manually populated, Lattes suffers from lack of standardization and typos. To deal with these problems we have used dictionarization [3] and approximate string matching strategies [4]. Subsequently, we have enriched the database with third-party information on research productivity related to journal and conference academic full papers. More precisely, we enriched our database with the following data:

1. *Impact Factor*: We have used the well-known Thompson's Journal Citation Reports (JCR) and Scopus's Scimago Journal Rank (SJR) impact factors.
2. *Citations*: We have considered citations of two different sources, namely Microsoft Academic Search (academic.research.microsoft.com) and Google Scholar (scholar.google.com).
3. *Indices*: We also have looked into how programs behavior face publication counts as well as h- and g-index – the indices have been calculated based on Google Scholar and Microsoft Academic citation counts.
4. *CAPES Qualis*: CAPES Qualis (or Qualis for short) is a set of criteria which CAPES uses to assess the Brazilian scientific production. From time to time, a new version of Qualis ranking is released assigning one of the following weights to each publication venues: A1, A2, B1, B2, B3, B4, B5, and C. In Qualis ranking system, A1 is the highest weight while C is unvalued. In order to compare only numerical measurements, numeric values have been assigned to each of the Qualis weight, namely: A1 = 100, A2 = 85, B1 = 70, B2 = 50, B3 = 20, B4 = 10, B5 = 5, and C = 0. Note this mapping from weights to numerical values are defined in the CAPES Computer Science Report. Information about the current Qualis classification is accessible online (qualis.capes.gov.br/webqualis).

### Analysis

Once we had all the above information obtained/derived we started the analysis itself. The analysis have followed two different lines: (i) productivity assessment and (ii) academic social network. In the former, we presented views of the programs from different perspectives and rank the programs accordingly. In the latter, we discussed the evolution of the Brazil's research productivity as a whole.

For a given graduate program, we present its performance from, e.g., the JCR, SJR, and Qualis perspectives. We show its citation count, h- as well as g-index for both Microsoft Academic Search and Google Scholar, and discuss how the results of its assessment may vary when the perspective changes. Concerning social networks, we have considered programs as nodes and drawn their collaboration also based on papers' coauthorship.

The algorithm used to identify all co-authors of a publication is based on the comparison between publication titles obtained from researchers/programs [5]. Because of inconsistencies in filling in information in Lattes, the comparison of any two publications is made through an approximate string matching between titles of papers. In other words, two papers are considered the same if their titles are at least 90% similar. The similarity between them was measured using the Levenshtein Distance [6]. It is important to note that the co-authorship between the programs 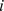 and 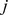 is referred to coauthorship between professors associated to graduate programs 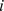 and 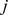, respectively. Professors associated with two or more programs are not taken into account in our analysis. A manually curated dataset was produced to evaluate the parser and the deduplication technique. This dataset contains information about 36 researchers and 620 publications. More than 99% of the fields were correctly parsed and the accuracy of the coauthorship identification was above 99% (with specificity above 99.9%, sensibility above 88% and F1 score about 94%).

## Results

In this section, we present results on program rankings and, subsequently, on academic social network.

### Program Ranking

Table 2 shows the programs numbered according to CAPES' reports. (I.e., programs 1 to 7 in Table 2, Column 1 correspond to the programs listed in Table 1.) They are ranked by the various metrics described previously, namely: Microsoft Academic Search citation count (MS CC) and Google Scholar citation count (Scholar CC) as well as h- and g-index based on them, impact factors (JCR and SJR), and Qualis considering the bibliographic production from 2004 to 2009. Table's values present the ranking position of a program for each of those metrics. It is worth noting that the positions of a program may greatly vary depending on the ranking being used.

**Table 2:** Brazilian CS graduate programs ranked using different metrics.

CAPES	MS	Scholar	JCR	SJR	Qualis	MS	MS	Scholar	Scholar	Pubs.	Best	Worst	Median
Prog. #	CC	CC				h-index	g-index	h-index	g-index	count	rank	rank	rank
1	2°	2°	13°	11°	10°	5°	6°	6°	5°	6°	2°	13°	6°
2	1°	1°	2°	1°	4°	3°	2°	3°	3°	7°	1°	7°	2.5°
3	5°	6°	4°	2°	5°	22	26°	20°	26°	4°	2°	26°	5.5°
4	6°	7°	10°	5°	2°	27°	28°	31°	32°	5°	2°	32°	8.5°
5	3°	3°	5°	3°	6°	23°	24°	26°	27°	2°	2°	27°	5.5°
6	4°	4°	3°	4°	8°	17°	15°	18°	19°	12°	3°	19°	10°
7	18°	11°	7°	6°	7°	37°	36°	35°	36°	9°	6°	37°	14.5°
8	11°	8°	8°	15°	12°	14°	17°	13°	15°	13°	8°	17°	13°
9	14°	15°	1°	13°	3°	24°	22°	27°	23°	34°	1°	34°	18.5°
10	13°	17°	12°	7°	9°	9°	12°	11°	13°	14°	7°	17°	12°
11	12°	12°	14°	9°	14°	8°	14°	9°	14°	17°	8°	17°	13°
12	10°	9°	9°	16°	13°	4°	4°	8°	4°	31°	4°	31°	9°
13	23°	24°	15°	17°	21°	25°	19°	22°	20°	32°	15°	32°	21.5°
14	9°	10°	25°	18°	17°	7°	7°	4°	7°	11°	4°	25°	9.5°
15	25°	25°	31°	33°	29°	28°	31°	24°	28°	28°	24°	33°	28°
16	27°	33°	17°	29°	30°	19°	16°	29°	25°	36°	16°	36°	28°
17	26°	26°	21°	23°	22°	32°	27°	34°	31°	26°	21°	34°	26°
18	19°	21°	11°	10°	18°	31°	32°	32°	35°	21°	10°	35°	21°
19	17°	16°	24°	8°	11°	15°	18°	15	16°	19°	8°	24°	16°
20	28°	27°	16°	22°	25°	35°	37°	37°	37°	18°	16°	37°	27.5°
21	21°	19°	27°	24°	23°	21°	29°	21°	24°	16°	16°	29°	22°
22	34°	34°	26°	28°	34°	30°	34°	28°	33°	33°	26°	34°	33°
23	24°	28°	22°	26°	26°	13°	11°	19°	18°	25°	11°	28°	23°
24	20°	20°	20°	20°	19°	12°	21°	10°	21°	10°	10°	21°	20°
25	7°	5°	6°	12°	1°	2°	5°	2°	2°	3°	1°	12°	4°
26	16°	18°	23°	21°	20°	10°	8°	14°	10°	20°	8°	23°	17°
27	29°	31°	18°	25°	15°	6°	9°	7°	9°	22°	6°	31°	16.5°
28	22°	22°	19°	19°	16°	11°	10°	5°	8°	29°	5°	29°	17.5°
29	15°	14°	30°	14°	24°	1°	1°	1°	1°	1°	1°	30°	7.5°
30	35°	35°	35°	31°	28°	29°	30°	30°	29°	23°	23°	35°	30°
31	32°	30°	28°	32°	36°	33°	23°	33°	22°	37°	22°	37°	32°
32	8°	13°	34°	30°	27°	20°	3°	25°	6°	8°	3°	34°	16.5°
33	30°	23°	37°	36°	31°	18°	25°	12°	17°	15°	12°	37°	24°
34	31°	32°	33°	27°	35°	16°	13°	17°	11°	24°	11°	35°	25.5°
35	36°	36°	32°	35°	33°	34°	33°	36°	34°	30°	30°	36°	34°
36	33°	29°	29°	34°	32°	26°	20°	16°	12°	27°	12°	34°	28°
37	37°	37°	36°	37°	37°	36°	35°	23°	30°	35°	23°	37°	36°

**Figure 2:**
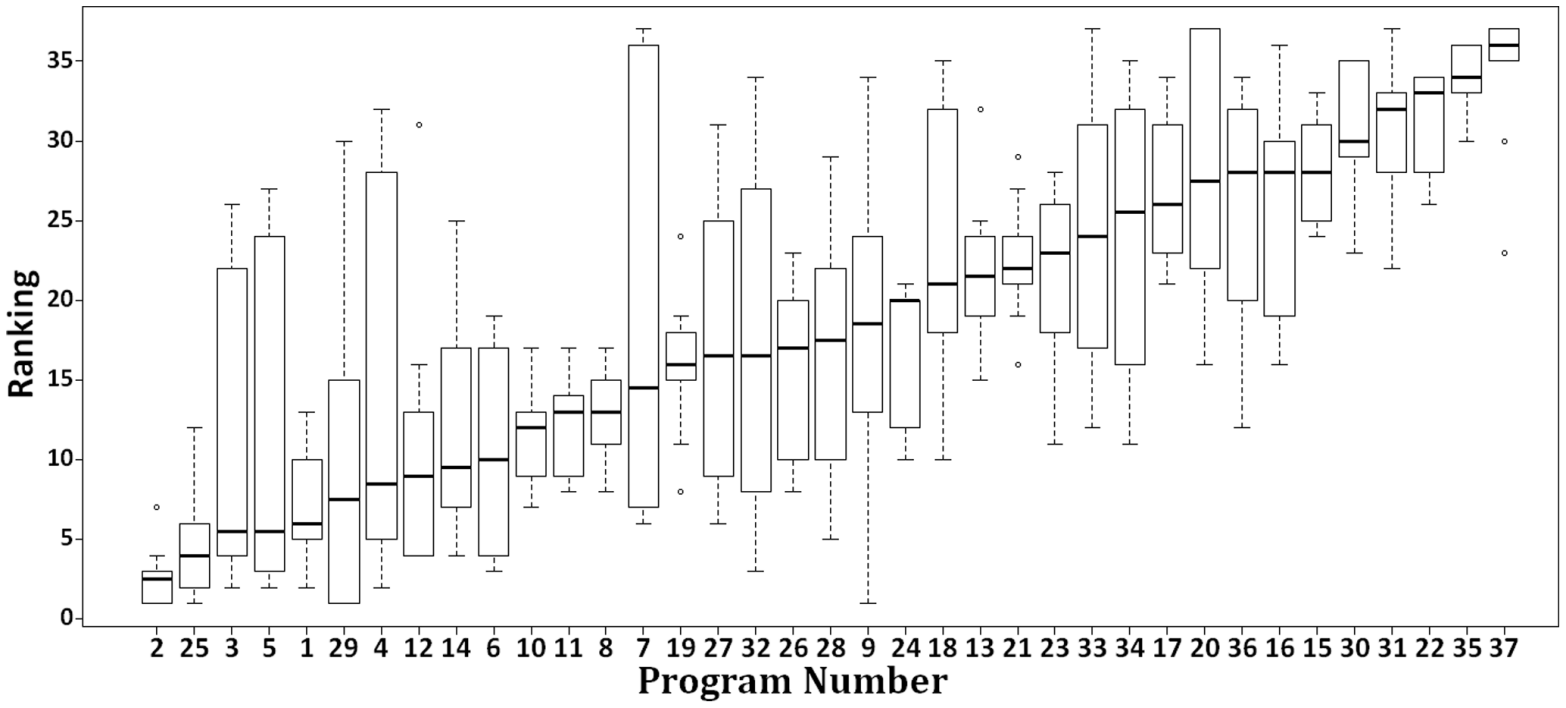
Boxplots of the programs' positions for the various rankings and evolution of programs in time.

**Figure 3:**
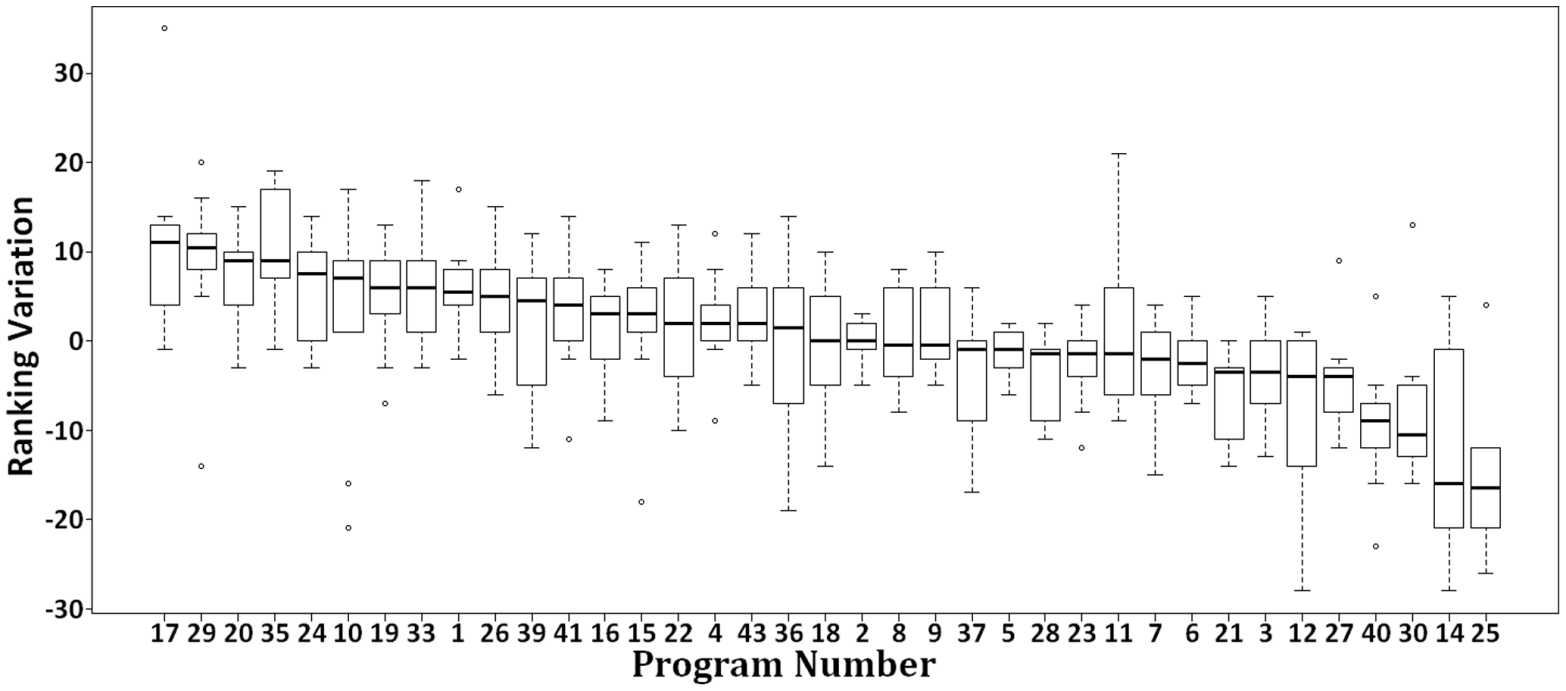
Difference between the rankings in the triennia 2007–2009 and 2004–2006.

Figure 2 and 3 illustrate this dynamic as well, i.e., it summarizes how the different rakings affect the programs' positions (Figure 2) and also shows how programs evolved over time (Figure 3). In these figures, the programs in axis 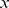 are sorted by the *median rank* metric.

Observe, for instance, that program 7's position ranges from 6th to 37th (Figure 2 and Table 2) and the median difference in the program 7's positions between the triennia 2007–2009 and 2004–2006 is -2, i.e., program 7 is better ranked in the triennium 2007–2009 (Figure 3). Besides, along the triennia, program 17's median ascended 11 positions and programs 2 and 18 have kept their median constant (Figure 3). All in all, programs 2, 25, and, 5 presented the lowest median in both triennia, i.e., 2.5, 4, and 5.5, respectively. (Figure 2). This concise representation allows us to observe the evolution (or not) of a given program in the context of Brazil.

**Figure 4:**
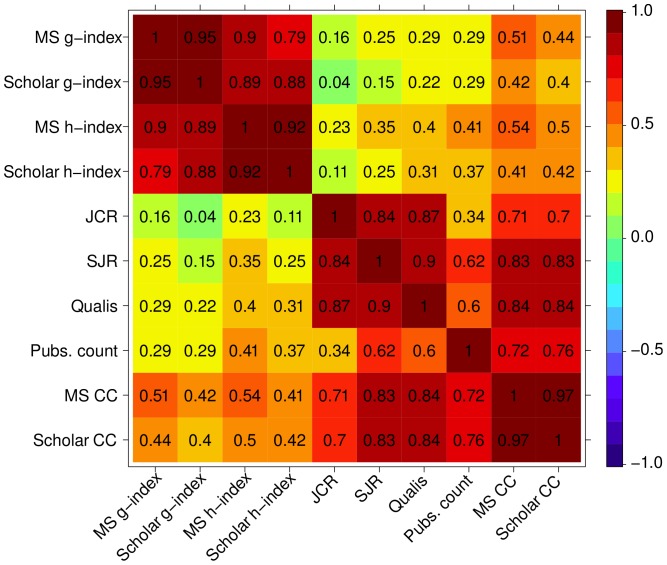
Correlation among the rankings.

We have also measured the correlation between the various rankings (Figure 3) and their correlation face the median ranking value (Figure 4). We highlight the correlation among three groups: (i) MS g-index, Scholar g-index, MS h-index, Scholar h-index; (ii) JCR, SJR, Qualis; and (iii) Pubs. count, MS CC, and Scholar CC.

According to Figure 4, the most representative rankings are the ones based on citations, namely Scholar CC and MS CC; both exhibiting a correlation greater than 90%. In fact, Scholar's correlation coefficient was slightly higher than MS's, reason why we have employed the Scholar CC ranking later in the next section as base for the comparison with network metrics.

### Network Analysis

So far in this paper we have shown how traditional productivity metrics affect program ranking. In this section we move away from these traditional metrics and start to look at the academic social network formed by the programs and the way they collaborate among themselves.

#### Network Formation

More specifically, we carried out a Social Network Analysis (SNA) over the academic social network made up by the programs. This is a particular type of social network in which the *nodes* represent the programs and the *edges* indicate that the programs (i.e., one or more of their professors) collaborated and published at least one paper together. Collaboration networks have been widely analyzed [7], as these studies disclose several interesting features about academic communities that comprise them.

Here we have built two sorts of collaboration networks, namely *undirected network* (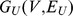) and *directed network* (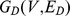); both of them having programs as the set of vertices (or nodes) 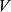 of a graph. In the former, an edge exists between two nodes if the programs they represent have published at least one paper together. In the latter, a directed edge 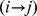 exists if programs 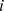 and 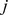 have coauthored a paper and the paper's first author is affiliated to program 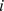. Here we assume the first author is the paper's main author and the other authors are researchers who supported her/him in the work. I.e., the set of edges 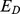 from 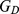 potentially maps the “needs help from” relationship between researchers. Regarding this particular network, it is worth pointing out that we are only considering papers in which the first author is a professor affiliated to a Brazilian CS program.

#### Network Metrics

Now we describe the network metrics we use to infer the productivity of the programs. As we will see, through these metrics we were able to find out clusters of programs and, more importantly, to highlight the program's roles in the CS production in Brazil.

#### Centrality in the network: 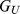


Node centrality is performed by using three metrics: degree (Cnt.Deg), betweenness (Cnt.Bet), and closeness (Cnt.Clos) centralities [8]. These metrics aim at identifying nodes that are strategically situated within the network's topology. A strategic location in a network may indicate that a node has a higher influence or even hold the attention of nodes that occupy positions that are not as socially relevant as its.

#### Clustering Coefficient of the network: 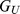


The clustering coefficient (Cl.Coef) of a vertex 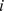 is regularly used to measure how clustered a group of vertices is [9]. In the case of 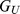, a low clustering coefficient likely means that the program has a wide network of collaborations, not being limited by geographical or any other constraints.

#### PageRank of the network: 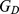


We have used the Google's PageRank algorithm to identify important nodes in directed networks by recursively transferring a node's importance to other nodes that the former “considers” important [10]. In the case of 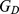, we apply the PageRank algorithm to identify those programs which are more requested for help in papers.

**Figure 5:**
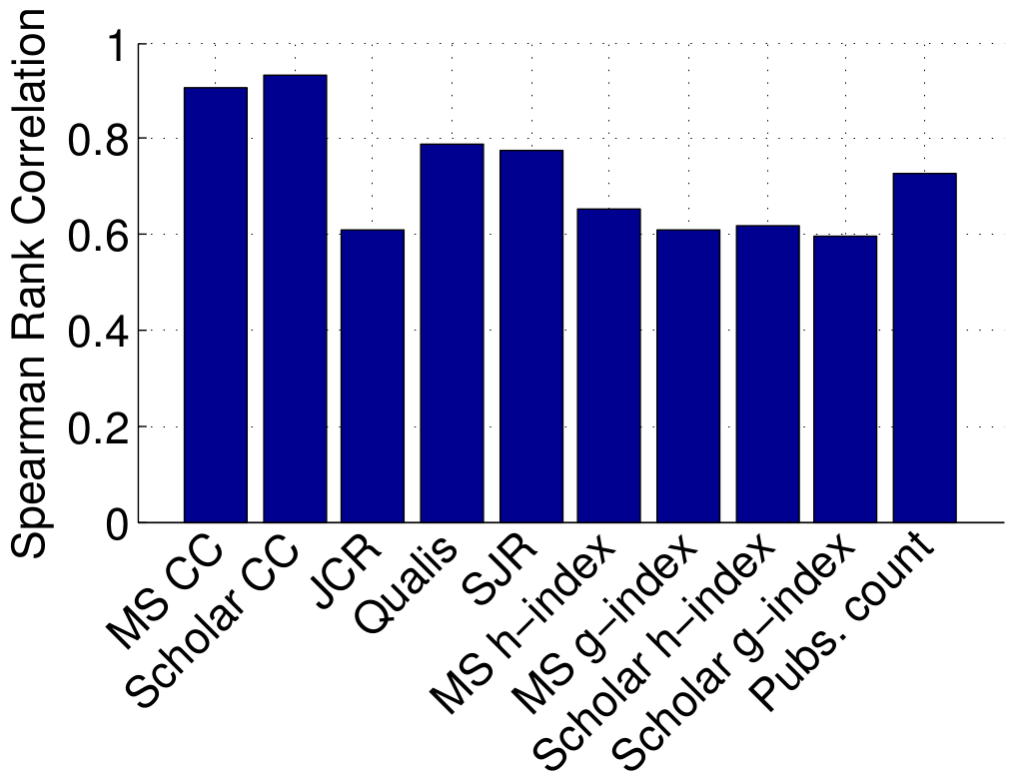
Spearman's correlation between each ranking and its median value.

**Figure 6:**
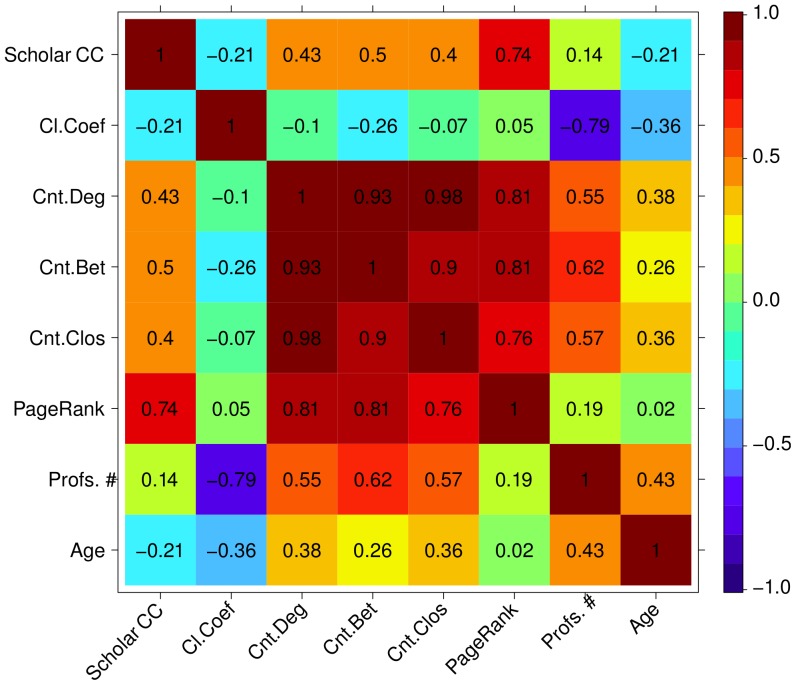
Spearman's Rank Correlation for different metrics.

In Figures 5 and 6, we show the two coauthorship networks among graduate programs we construct in this paper. Node colors represent the geographic region which the program is located, namely north (blue), northeast (red), central-west (purple), southeast (green), and south (gray). Node sizes are proportional to their degrees for 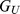 and to their PageRank for 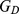. Again, we label the top seven programs according to CAPES (see Table 1 and Column 1 of Table 2). These graphs are drawn using a force-direct layout algorithm (FDLA) [11], which tries to minimize the number of crossing edges. In other words, this layout highlights communities of nodes, i.e., nodes with a high number of common neighbors are placed closer.

First, it is worth noting that the top programs are clearly visible in both networks, i.e., they have high values of degree and PageRank, what indicates that these two network metrics are able to highlight them. Moreover, observe that there is no clear community or cluster among the programs, although there is a slight tendency of geographically closer programs to collaborate more. The geographic assortativity [12] considering the region of each program is 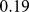. On the other hand, the degree assortativity is negative for both networks, indicating that there is also a small tendency of programs with different degree magnitudes to connect more. This is expected, since it is a common practice of smaller programs to collaborate with bigger ones. This is because a significant part of the professors of the former have graduated in the latter. In Table 3 we describe these and other characteristics of the networks 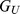 and 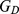.

**Table 3:** Characteristics of the networks.

Metric	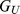	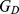
Density	0.25	0.18
Average Degree	11.4 (7.3)	in: 8.2 (5.9), out: 8.2 (5.4)
Clustering Coefficient	0.56	-
Diameter	3	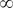
Average Distance	1.9	1.9
Degree Assortativity	−0.23	−0.27
Region Assortativity	0.18	0.18

We believe that the aforementioned node's features are able to capture a great part of the programs collaboration dynamics. While the centrality metrics indicate the “importance” of the program in the network, the clustering coefficient shows how broad are the node's connections. Additionally, the PageRank metric is able to capture a certain degree of hierarchy among the programs, pointing which program receives more “help” requests.

In order to further investigate these features' potential to discover knowledge, we rank the programs according to each of the aforementioned network metrics, and three other metrics, i.e., the program's age, the program's size (*Profs. #*) and the program's Scholar CC productivity index. We show in Figure 5 the Spearman's Rank Correlation 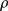 among these ranks. If, for instance, the correlation 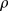 between the rank generated by the degree centrality and the Scholar CC is 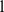, then the degree centrality generates the same rank of programs the Scholar CC generates. If 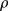 is 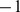, then it generates the complete opposite rank.

First, note how the network metrics have positive rank correlation with the Scholar CC. Additionally, PageRank is the metric most correlated with the Scholar CC, what corroborates to our assumption that in papers involving different programs, the first author usually seeks for aid in other programs, creating the so called “needs help from” hierarchy in the network.

#### Network-based Classification

**Figure 7:**
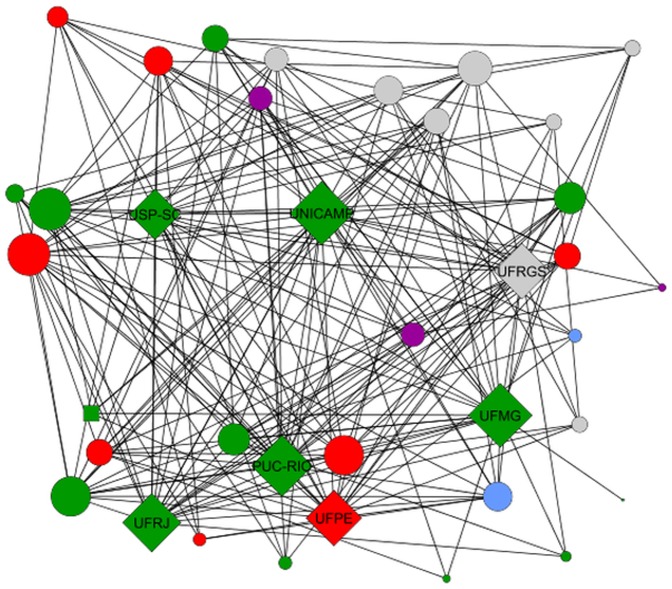
Undirected co-authorship network belonging to 37 Brazilian CS graduate programs. The programs are represented by nodes, the co-authorships by edges, and node sizes are proportional to their degree.

**Figure 8:**
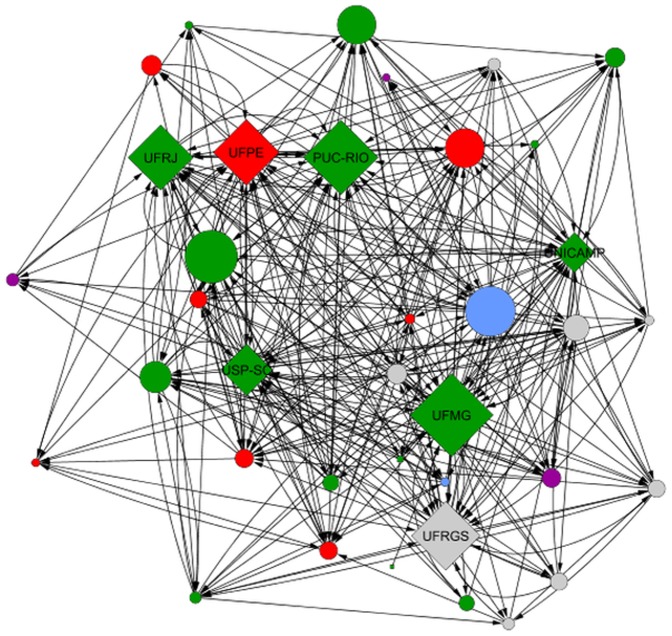
Directed co-authorship network belonging to 37 Brazilian CS graduate programs. The programs are represented by nodes, the co-authorships by edges, and node sizes are proportional to their PageRank.

As we observed in Figures 7 and 8, simple metrics are able to highlight the most important nodes in the network. However, observe how these metrics produce very different results and fail to separate the top 7 programs according to CAPES from several other nodes, which have apparently similar importance in the network.

Thus, in order to verify if network metrics are able to clearly separate these top programs from the rest, we analyze the principal components of the feature matrix formed from these metrics. Principal Component Analysis (PCA) [13] is a widely used statistical technique for unsupervised dimension reduction. It transforms the data into a new coordinate system such that the greatest variance is achieved by projecting the data into the first coordinate, namely principal component, the second greatest variance achieved into the second coordinate, the second component, and so on.

**Figure 9:**
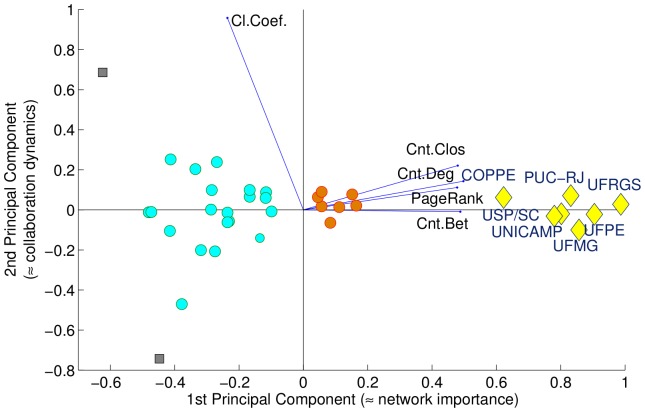
The first two principal components of the matrix formed from the network features.

In Figure 9, we show the first two principal components of the matrix formed from the network features. These two components account for approximately 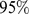 of the variation. It is fascinating that these new dimensions are able to clearly cluster the Brazil's top programs according to CAPES (labeled in the figure). Note that the first dimension, which accounts for approximately 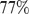 of the variation, is more related to the node importance in the network, since the component coefficients of the centrality metrics and the PageRank are significantly positive. On the other hand, the second component, which accounts for approximately 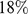 of the variation, is more related to the clustering and collaboration dynamics of the programs. Note that it is able to discriminate well programs located in the left far side of the figure, indicating that a program should also avoid a collaboration strategy that leads to either very high or very low values for the clustering coefficient. To illustrate that, consider the two most far points in this dimension, both marked with a square symbol. While the one with the most positive value has a degree of 6 and a clustering coefficient of 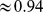, the most negative one has a degree of 1 and a clustering coefficient of 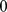 (by definition).

#### Deconstructing the Collaborations

We have shown so far that network metrics have significant correlation to research productivity in our context. In this section, in turn, we go further in this analysis by looking into the reasons why an edge remains persistent (kept alive) over the years. Again, we have considered the period between 2004 and 2009, divided into two triennia (i.e. 2004–2006 and 2007–2009). We consider that an edge 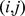 is persistent if program 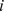 collaborated with program 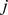 in both triennia. Otherwise, we call this edge non-persistent.

**Figure 10:**
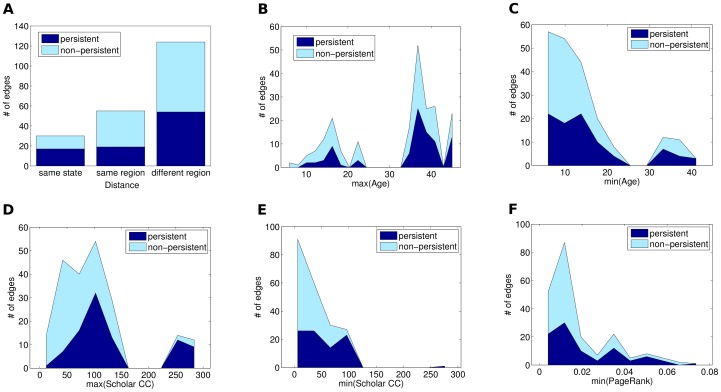
The number of edges which are persistent and non-persistent according to various metrics.

In Figure 10, we show the total number of edges that are persistent and non-persistent grouped by six different metrics, namely distance, max(Age), min(Age), max(Scholar CC), min(Scholar CC), and PageRank. More precisely, in Figure 10A, we group the edges 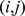 by the geographic distance between nodes 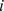 and 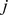. Note that the fraction of non-persistent edges grows significantly as the distance grows, what indicates that distance is a determinant factor for an edge to persist or not.

Figure 10B and Figure 10C, in turn, shows that the program's age also influences the edges persistence. They show, respectively, the age of the older – max(Age) – and the younger – min(Age) – node of the edges. Note that the proportion of persistent edges is significant only when max(Age) is higher than 30. Also, note that as min(Age) grows, the proportion of persistent edges grows as well, indicating that whenever the edge is between two old (well-established) nodes, the edge is more likely to be persistent.

In addition to nodes distance and age, we also studied how edge persistence varies as a function of nodes productivity. In Figure 10D and in Figure 10E, we show the number of persistent and non-persistent edges grouped by the Scholar CC value of the most – max(Scholar CC) – and the least – min(Scholar CC) – productive node that comprises edges. First, note that while it is very unlikely to have a persistent edge when max(Scholar CC) is very low, it is very unlikely to have a non-persistent edge when max(Scholar CC) is high (Figure 10D). Besides, from Figure 10E, we can note that the proportion of non-persistent edges drops significantly as min(Scholar CC) grows. All these observations suggest that the node productivity is a key factor for the persistence of its edges.

Now, in Figure 10F, we consider a network metric, i.e., we show the number of edges grouped by the minimum PageRank value (min(PageRank)) of their nodes. Observe that the proportion of non-persistent edges drops significantly as the min(PageRank) of the node grows. This suggests that edges formed from nodes with high PageRank values are likely to be persistent. In other words, when programs which are frequently providing “help” to other programs (i.e. high PageRank programs) collaborate among themselves, it is very likely that the “help” will come in a bidirectional way, reinforcing the collaboration and, as a consequence, the edge persistence.

Finally, observe how the histograms of Figures 10B, 10C and 10D are bimodal, showing two well defined masses of data. This fact corroborates with the cluster analysis we showed in Figure 9.

All in all, it looks like that the edge formation process is governed by at least three processes that splits the edges (and the programs, as we see in Figure 9) into groups with different characteristics. We conjecture that these edge creation processes are the following:

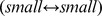
. Occurs when new or low-productive programs collaborate among themselves. The collaboration may have started, for instance, when two ex-colleagues graduated together and kept collaborating after they started to work for different new programs. These edges are more likely to not persist.

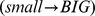
. Occurs when a new or low-productive program seeks a collaboration with well-established ones. These edges are created when, for instance, a professor of a new program graduated in an established one and continued to collaborate with her/his former advisor. These edges are either like to persist or not.

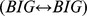
. Occurs when well-established programs collaborate among themselves. It is common that experts from well-established programs are well known by the academic community and seek each other's “help” in a bidirectional collaboration. These edges are more likely to persist.

## Discussion

Recently, the number of works focused on research productivity assessment has grown considerably (e.g. [5], [14]–[18]). Most of them, relies on metrics such as Impact Factor [16], [19], h-index [14], and citation count to assess productivity in a certain area of research. Duffy, Jadidian, and Webster [17], for instance, carried out an evaluation of academic productivity within psychology. Their work has been based on h-index, citation counts, and author-weighted publication counts. Further, they have analyzed the impact of the gender and tenure on researchers' productivity.

Martins *et al.* [18], in turn, have assessed the quality of conferences based on citation count. They have also pointed out the need for new metrics and come up with some of them exclusively tailored to assess conferences.

There are also works that combine SNA and the CS field (e.g. [20]–[22]). Among them, Menezes *et al.* [20] analyzed the CS research productivity in different regions of the world (Brazil, North America, and Europe) using collaboration networks. They presented the evolution of CS subfields for the period 1994–2006 and the inter-relationship between the CS subfields. They have also discussed the research productivity in each world region; and contrasted the regional networks' idiosyncrasies.

Franceschet [22], in turn, collected data from the DBLP CS Bibliography [23] to study coauthorship networks and analyzed academic collaboration in CS. Franceschet have shown that the collaboration level in CS papers is rather moderate compared to other fields. Their results also indicates that conferences can communicate results quicker, while journals can make relationships stronger.

Like ours, some works have also narrowed even further their object of study and focused on the Brazil's CS research productivity. For instance, Laender *et al.* [2] have assessed the excellence of the top Brazilian CS graduate programs. They have contrasted Brazilian programs against reputable programs in both North America and Europe and conclude that the CS field in Brazil has reached the maturity. This study has been based on recent data from DBLP.

Digiampietri and Silva [24], in turn, introduced a framework for social network analysis and visualization that allows users to access relevant information about research groups using web-available data. The framework searches curricula in Lattes, extract relevant information, identify the relationships among authors, and then builds a social network. In the work, they have used the field of CS in Brazil as a case of study.

Finally, Figueiredo and Freire [25] have presented a study of the Brazil's CS academic social network based on the DBLP. In this network, they have noticed the existence of super peers, i.e., that a small number of nodes presented a very high degree (some researchers collaborated with many other researchers), while the great majority of nodes had a lower degree. They have also come up with a metric, namely *degree-cut-weight*, to classify individuals in collaborative networks, where the importance of a node in a group is proportional to the intensity of their relationships with nodes from another group. They then applied this and other metrics to rank graduate programs and compared the results with CAPES ranking.

Likewise the aforementioned work [25], our work studies the Brazil's CS academic social network, as well. However, our approach is different. See, first we collect our dataset from Lattes, to our knowledge, the most reliable base of the Brazilian scientific production. Second, while Figueiredo and Freire aim at evaluating CS programs using three metrics (namely degree-cut-weight as well as number of publications and collaborators), our goal is to assess them from ten different perspectives based on quantitative ranking systems. Finally, different from [25], we isolate network metrics from quantitative metrics to show that network metrics alone can spot the most productive CS programs in Brazil.

## Conclusions

Research productivity assessment is important because it allows allocating the limit funds to foment research activities in a meritocratic way. However, the fact that the assessment involves both qualitative and quantitative analyses of several characteristics – most of them subjective in nature – turns this assessment challenging. Besides, depending on the metrics used to carry out the assessment, results change.

In this work, we aim at presenting an X-ray of Brazilian CS graduate program's research productivity. To be precise, we have shown views of the programs from different perspectives such as h- and g-index as well as citation counts. To do that, we have explored mainly two characteristics: (i) the intellectual productivity and (ii) the academic social network for the period between 2004 and 2009. The results indicate that programs better located in the network topology are more productive. We believe that the obtained results are paramount for assessing academic performance, getting current collaborations stronger, and pointing out new partnerships among programs.

Despite our study was able to draw precise conclusions, future directions are still possible. For instance, one could adapt our methodology and thus apply it to assess other Graduate Progress, whether in Brazil or not. Besides, our methodology could be improved by also taking into consideration the roles that funds and governmental incentives play in the scientific production.
